# Books and Pamphlets Received

**Published:** 1886-07-20

**Authors:** 


### BOOKS AND PAMPHLETS RECEIVED.

*A Reference Hand book of the Medical Sciences*; embracing the entire range of Scientific and Practical Medicine and Allied Sciences, by various writers. Illustrated by chromo-lithographs and fine wood engravings. Edited by Albert H. Buck, M.D., New York City. Vol. II. New York: William Wood & Co., 56 and 58 LaFayette Place. 1886.

The scope of this elaborate work, as set forth in our notice of the first volume, is more fully comprehended by an examination of the articles in this second volume, which extend from acute nasal catarrh to the affections of the eye, terminating with tumors of this organ. Many of the papers indicate great research, and a large number are illustrated in the best style of art, in its several departments. We are struck with the faithfulness of the woodcuts which illustrate one of Professor Gaston's articles, and with the excellence of the several colored engravings. With the exception of one from Toronto and four from Montreal, Can., the contributions are by residents of the United States, and we observe among the Southern cities that Baltimore furnishes six of the contributions; New Orleans three, Atlanta two, Augusta one, and Charleston one. Of the last named city, Dr. F. Reyse Porcher furnishes a very practical and most useful paper upon Emmenagogues. Dr. Eugene Foster, of Augusta, contributes a highly instructive and eminently practical article on Dengue Fever, and another paper giving a timely lesson on Enemata. Brief and terse notices of Cervico-bronchial and Cervico-occipital Neuralgia are given by Dr. James B. Baird, of this city. Three distinct and elaborate essays on the Obstructions of the Gall duct, and the different operative procedures for their relief, from the pen of Dr. J. McF. Gaston, of Atlanta, appear under the headings of Cholecystostomy, Cholecystostomy, and Duodeno-cholecystostomy, which must be read to be properly understood and duly appreciated.

